# Polish national nature-based mental health prevention programme

**DOI:** 10.1192/j.eurpsy.2026.10754

**Published:** 2026-07-31

**Authors:** M. Gawrych

**Affiliations:** Institute of Psychology, The Maria Grzegorzewska University, Warsaw, Poland

## Abstract

**Introduction:**

A holistic approach to health, including mental health, entails the consideration of multiple perspectives on well-being and illness—ranging from the intrapsychic level, through interpersonal and community dimensions, to environmental factors. In the context of a global and complex mental health crisis, coupled with the lack of unequivocally effective methods for preventing mental disorders, the search for simple, low-cost, and universally accessible interventions has become a public health priority.

**Objectives:**

This paper presents a preliminary conceptual framework for a culturally grounded, scalable, and accessible mental health prevention strategy tailored to the specific needs and systemic realities of the Polish context. While potentially suitable for systemic implementation, the proposed approach requires further empirical research and validation.

**Methods:**

The Polish strategy for nature-based prevention programmes was developed through an extensive review of the literature, encompassing guidelines and policy documents from European countries—particularly those with temperate climates—as well as historical sources and empirical research findings. In addition, reports and best practices from European and international organisations specialising in mental health and nature-based therapeutic interventions were examined.

**Results:**

he nature-based prevention programme encompasses both the general population and vulnerable groups, including those with clinical needs, and is intended for implementation across diverse contexts—educational (school-based), occupational (workplace), and healthcare settings. The poster will characterise diverse forms of nature-based interventions, including those targeted at specific clinical groups, and will propose an algorithm for matching intervention methods to individual needs, in line with a patient-oriented framework.

**Conclusions:**

A national prevention strategy grounded in scientific evidence and clinical practice data may serve as a complementary, supplementary, or supportive method for promoting the health of the general population, as well as aiding the recovery process of individuals with mental disorders.

**Disclosure of Interest:**

None Declared

